# Exploring miniaturized EEG electrodes for brain-computer interfaces. An EEG you do not see?

**DOI:** 10.14814/phy2.12362

**Published:** 2015-04-06

**Authors:** Martin G Bleichner, Micha Lundbeck, Matthias Selisky, Falk Minow, Manuela Jäger, Reiner Emkes, Stefan Debener, Maarten De Vos

**Affiliations:** 1 Neuropsychology Lab, Department of Psychology, European Medical School, University of OldenburgOldenburg, Germany; 2 Cluster of Excellence Hearing4all, University of OldenburgOldenburg, Germany; 3 EASYCAP GmbHInning, Germany; 4 Neurosensory Science, University of OldenburgOldenburg, Germany; 5 Institute of Biomedical Engineering, Department of Engineering, University OxfordOxford, UK

**Keywords:** Ear EEG, miniaturized, P300 speller, ton-electrodes

## Abstract

Electroencephalography (EEG) allows the study of the brain–behavior relationship in humans. Most of what we have learned with EEG was through observing the brain–behavior relationship under well-controlled laboratory conditions. However, by reducing “normal” behavior to a minimum the ecological validity of the results can be limited. Recent developments toward mobile EEG solutions allow to study the brain–behavior relationship outside the laboratory in more natural situations. Besides mobility and robustness with respect to motion, mobile EEG systems should also interfere as little as possible with the participant's behavior. For example, natural interaction with other people could be hindered when it is obvious that a participant wears an EEG cap. This study evaluates the signal quality obtained with an unobtrusive solution for EEG monitoring through the integration of miniaturized EEG ton-electrodes into both a discreet baseball cap and an individualized ear piece. We show that such mini electrodes located at scalp and ear locations can reliably record event related potentials in a P300 brain–computer–interface application.

## Introduction

Over the past decades, noninvasive brain activity recording with electroencephalography (EEG), functional magnetic resonance (fMRI) and other techniques increased our knowledge of the brain–behavior relationship. A disadvantage is that natural behavior is eliminated to a large extent in well-controlled laboratory settings, due to motion intolerance of these noninvasive recording procedures. While fMRI is not portable, research-grade EEG suffers from the limitation that gross movement degrades signal quality (Debener et al. 2012; De Vos and Debener 2014). Recent developments in mobile electroencephalography (EEG), however, promise the monitoring of brain activity during natural behaviors outside the laboratory environment (Debener et al. 2012; Gramann et al. 2014), which may improve the ecological validity of neuroscientific investigations (Baumeister et al. 2008). Unobtrusive, easy to use EEG technology would be desirable to achieve this goal.

Monitoring of mobile brain activity promises the translation of neuroscientific knowledge into clinical and daily life applications. Using mobile EEG, good quality single-trial event-related EEG signals were obtained while participants walked on a treadmill (e.g., Lin et al. (2014)) and even while participants walked freely outdoors with wireless, lightweight and fully head-mounted systems (Debener et al. 2012; De Vos et al. 2014a). Moreover, wireless EEG enables the study of brain activity in working environments (Wascher et al. 2014), it can provide diagnostic information (Grant et al. 2014), enables brain–computer interface (BCI) performance comparable to traditional laboratory EEG technology (De Vos et al. 2014b) and has a clear translational potential, in particular in the field of neurorehabilitation (Kranczioch et al. 2014). However, a major limitation of currently available systems is the clear visibility of the EEG electrodes, which are normally embedded into electrode caps. This prevents comfortable, continuous and near-invisible EEG recordings in natural environments and everyday situations.

Aiming to reduce the visibility of EEG recordings, miniaturized, wet electrode solutions were proposed (Nikulin et al. 2010). Due to the small size, these electrodes can be worn discretely at typical EEG scalp locations. The small surface of such mini electrodes might increase the electrodes-skin impedance (Nikulin et al. 2010), but this has no detrimental impact on the quality of the signals when high input impedance amplifiers are used (Ferree et al. 2001; Nikulin et al. 2010).

Others took the idea of miniaturized electrodes a step further and introduced the in-ear EEG recording concept (Looney et al. 2011, 2012). By means of individualized ear-fittings several electrodes were positioned in the outer ear canal and on the concha. In-ear EEG combines a number of practical advantages, such as easy and reliable application, discreet positioning, good user comfort and avoidance of hair washing (Looney et al. 2012). Moreover, a tight fit and the low weight of electrodes and cables minimizes motion artifacts (Debener et al. 2012; Looney et al. 2012). With in-ear EEG, event-related potentials (ERPs) were reported initially for a single case (Looney et al. 2011) and subsequently for two individuals (Looney et al. 2012). Kidmose et al. (2013) report plausible auditory (AEP) and visual evoked potentials (VEP) for three participants.

**Figure 1:**
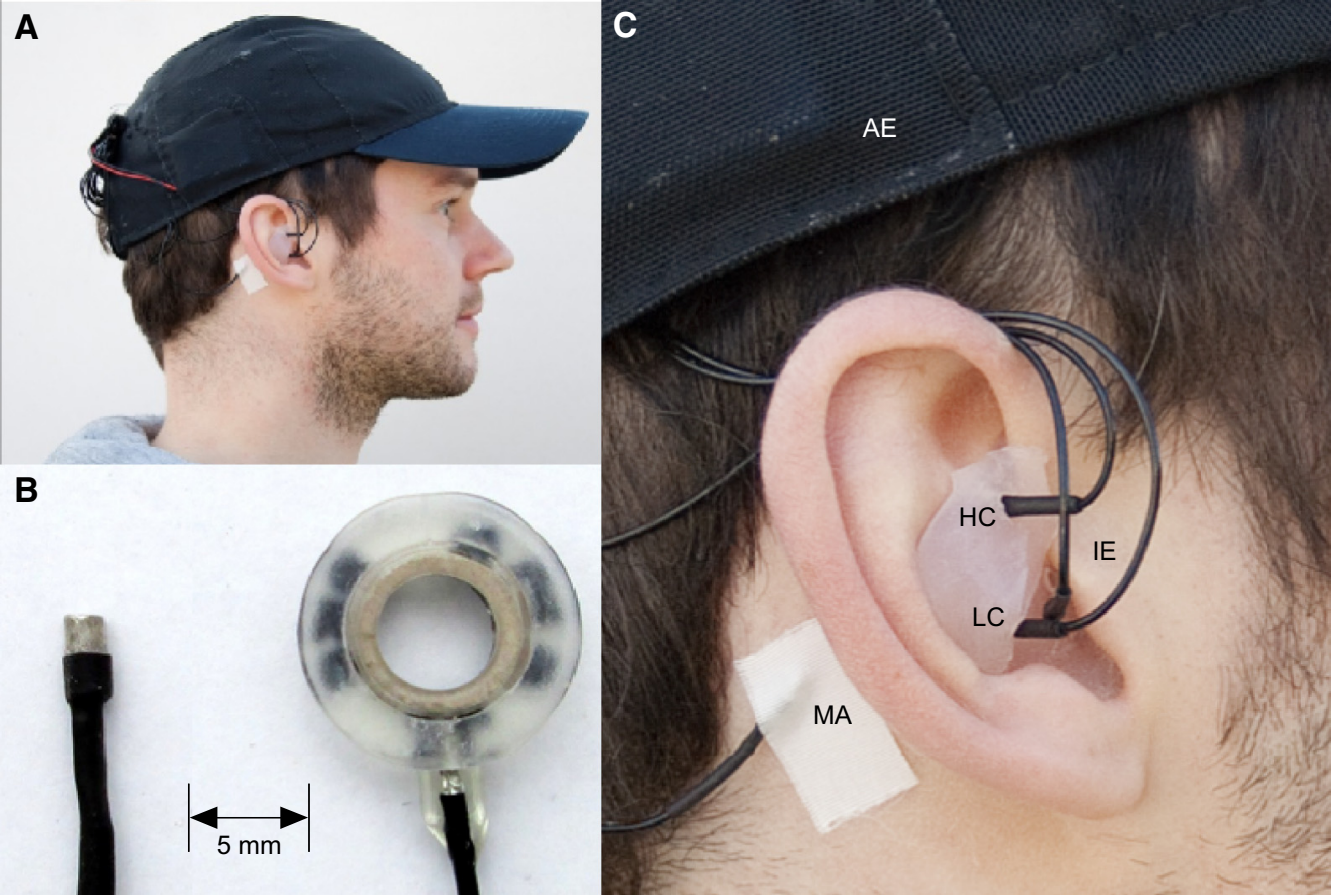
(A) Recording setup with baseball cap and individualized silicone ear piece. (B) Size comparison between mini electrode and a standard EEG ring electrode. (C) Close up of the ear piece. Electrodes were located in the high concha (HC), the low concha (LC), and the outer ear canal (IE). Reference electrodes were located above the ear (AE) and on the mastoid behind the right ear (MA).

Aiming toward concealed EEG recordings, we integrated identical miniaturized EEG electrodes into a baseball cap, into individualized silicone earpieces (Fig.1) and placed additional electrodes behind and above the ear. We investigated in a larger sample size if such a nearly invisible solution allows to reliably record those ERP signals that are traditionally used to operate a BCI. Specifically, we compared miniaturized ear electrodes with concurrently recorded EEG data using miniaturized scalp electrodes. With this set-up, participants performed a popular BCI matrix copy-spelling task (Farwell and Donchin 1988) as used in a previous study conducted in our laboratory using traditional EEG electrodes (sintered Ag/AgCl; De Vos et al. (2014a)). This enabled the comparison of BCI performance obtained with traditional versus miniaturized electrodes. Our main goal was to investigate whether recordings taken from locations in and around the ear can be used for ERP recording and BCI.

## Materials and Methods

### Participants

Twelve healthy individuals (6 female, age mean 26 ± 5.8) free of past or present neurological and psychiatric conditions participated in the study. The study protocol was approved by the ethics committee of the University of Oldenburg and all participants signed a written informed consent prior to participation.

### Data acquisition

EEG data were recorded from 13 sintered Ag/AgCl miniaturized (2 × 4 mm) ton electrodes (EasyCap GmbH, Herrsching, Germany; as shown in Fig.1) connected to a 16 bit BrainAmp amplifier (Brainproducts GmbH, Herrsching, Germany) at a sampling rate of 500 Hz. Eight electrodes were integrated into a baseball cap according to the 10–20 system (C3, Cz, C4, P3, Pz, P4, AFz and FCz). To accommodate different head sizes, two different baseball caps were used. AFz and FCz served as ground and reference electrode for online recording. One electrode was placed on the mastoid behind the right ear, and one electrode was placed directly above the right ear (see Fig.1). Three electrodes were integrated into individualized ear-pieces (see below); two electrodes were located in the concha and one in cranial direction in the outer ear canal. The recording sites in the ear were cleaned with alcohol prior to the recording. Good conductivity between skin and all electrodes was ensured using an abrasive electrode gel (ABRALYT HiCl, EasyCap GmbH). Electrode impedances were kept below 25 kΩ. For 6 participants, the impedance of one channel could not be lowered below 25 kΩ, these channels were excluded from further analysis. The average impedance was 9.9 kΩ (SD = 3.99) for the cap channels and 10.8 kΩ (SD = 3.83) for the ear channels. There was no significant difference in impedance between cap and ear channels (paired *t*-test, *t* (11) = −0.74, *P *= 0.48).

### Ear-piece

Individualized ear-pieces were produced for each participant several days prior to the EEG recording session by a certified laryngo-rhino-otology assistant (MH). Fitted was always the right ear, after inspection of the outer ear canal and the tympanic membrane. Where necessary participants underwent a professional ear-cleaning prior to the fitting. The material used for the ear-pieces was a two-component addition-curing silicone (Dreve Otoplastik GmbH, Unna, Germany). After fitting, three holes were drilled into each ear-piece to place the electrodes. A small dent of approximately 2 mm was left as a pocket to be filled with conductive gel.

### Paradigm and software details

Participants performed a visual P300 copy-spelling task using a matrix speller. BCI2000 (version 3.0.5) was used for stimulus presentation and online classification. All parameter settings were identical to those used by De Vos et al. (2014b). Briefly, a 6 × 6 spelling matrix was used containing letters, numbers, and the “space bar”. The participants had to copy spell one of a series of German sentences containing 19 symbols (letters, numbers and spaces); for example, HEINZ_MALT_53_PUDEL. Each participant performed one sentence during a training block and a new sentence during the following online block. During the online block, participants received feedback about the letter that was decoded based on the brain signals. Each letter in the matrix was highlighted (flashed) 24 times (12 times per row and 12 times per column). In contrast to De Vos et al. (2014b), the number of flashes was not reduced during the online block, but was kept identical for both blocks. The flash length was set to 125 msec with an inter-flash interval of 60 msec. The participant had to concentrate on the letter she wanted to spell and count silently how often this letter was highlighted. Between letters, a break of 2000 msec was included, allowing the participant to concentrate on the next letter. For the online feedback, step-wise linear discriminant analysis was used for classifier training using default BCI2000 parameter settings (i.e., all channels were used, the *P*-value for including features in the step-wise procedure was 0.1 and for excluding features it was 0.15) (Krusienski et al. 2008).

### EEG analysis

The offline analysis was performed with EEGLAB (Delorme and Makeig 2004) and MATLAB (Mathworks Inc., Natick, MA). The data from the training and online block were combined and filtered with a 0.3 Hz high-pass filter and a 20 Hz low-pass filter. Subsequently data were re-referenced. Note that the cap data were re-referenced to the mastoid channel (MA, Fig.1), a popular scalp EEG reference site, and the ear data were re-referenced to the above ear channel (AE, Fig.1). The reason for using different reference channels for the ear data was to provide a local, unobtrusive reference and avoid isolated electrodes wires.

Epochs to target letter flashes (attended) and nontarget flashes (unattended) were extracted (0–800 msec). Averaging across epochs resulted in ERP waveforms, which were computed for each electrode for the attended and unattended letters. The P300 was evaluated by calculating the difference between nontarget and target ERPs. Note that we performed no further preprocessing or artifact rejection for the offline analysis, aiming to evaluate the data similar to an online application. To quantify the condition effects between scalp and ear EEG data, the Hedges’ g effect size measure was computed between 0 and 800 msec using a moving window with a length of 100 msec. This measure is very similar to the more commonly used Cohen's d standardized mean difference effect size but includes a Bessel correction for variance estimation, which is more appropriate for small sample sizes.

For each participant and electrode the mean amplitude for the attended and unattended stimuli in the time window between 400 and 700 msec was computed and subjected to a repeated measures ANOVA comprising the factors electrodes (10 electrodes, i.e., six cap channels and four ear channels) and condition (attended, unattended). Significant effects were followed up with *t*-tests where appropriate.

To estimate the relevance of individual time bins and individual channels irrespective of statistical significance, as commonly done in BCI feature evaluation (Blankertz et al. 2011) single-subject point biserial correlation coefficients between target and nontarget trials were calculated (shown as unsigned *R*^2^). An equal number of target and nontarget trials was used for the analysis. Specifically, we used 100 random sub samples of the nontarget trials and computed the *R*^2^ value with the target trials. Afterward the results of the 100 repetitions were averaged to obtain the mean *R*^2^ value.

To compare our results to an earlier study we computed the information transfer rate (ITR). The ITR is a commonly used metric to evaluate and compare BCI performance over studies. It expresses the communication speed of a given system in bits of information that can be transmitted per minute. The ITR was computed as described in De Vos et al. (2014b).

The regular flashing of individual rows and columns generated a steady-state visual evoked response (SSVEP) at a frequency of 5.4054 Hz (185 msec inter flash interval). To estimate the strength of the SSVEP at ear and cap channels, we computed the log power of the SSVEP for all participants and channels. For each sequence of flashes, the signal was averaged in the time domain and the power was computed using the fast Fourier transform. For the frequency of interest (5.4054 Hz), a repeated measures one-way ANOVA was computed with electrodes as factor. A Greenhouse–Geisser correction compensating for violations of sphericity was applied where appropriate.

## Results

The ERPs at the classical recording sites PZ and CZ showed a clear SSVEP at around 5 Hz in response to the visual stimulation. In the time domain a clear P300 (400–700 msec) component in response to target letters was evident. Over all electrodes, there was a significant effect between attended and unattended stimuli (*F* (1, 11) = 34.85, *P *< 0.001) on the P300 amplitude. There was also a significant main effect of electrodes (*F* (9, 99) = 9,136, *P *= 0.001) and a significant interaction between electrodes and attention (*F* (9,99) = 11.80, *P *< 0.001). The follow-up paired *t*-tests revealed that there was a significant attention effect for all of the cap electrodes and for one of the ear electrodes (high concha, HC, *t* (11) = 5.851, *P *< 0.001, Bonferroni corrected). The amplitude in the attended condition was significantly higher for the cap channels compared to the ear channels (paired *t*-test *t* (11) = 4.453, *P *= 0.001).

The SSVEP log power was significantly different between electrodes as revealed by the repeated measures ANOVA (*F* (9, 99) = 14.75, *P *< 0.01). The post hoc analysis revealed that the log power for the average of the ear channels was significantly smaller compared to the average of the cap channels (*t* (11) = 4.94, *P *< 0.001).

**Figure 2:**
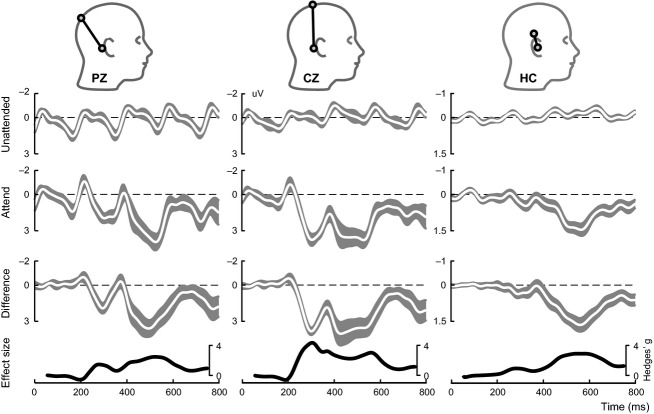
Average ERP trace (shown in white) for PZ, CZ and HC (ear) shown for unattended flashes (1st row), attended flashes (2nd row) and the difference wave (3rd row). The shaded gray area shows the standard error of the mean. Note the values on the *y*-axis of the ear electrode are half the size of the two cap electrodes. The heads indicate the position of the respective recording electrodes (black circle). The effect size over time is given as Hedges’ g (bottom row)

The morphology of the ERPs at CZ, PZ, and HC appeared similar to some extent (Fig.2). They all showed a clear positive deflection between 400 and 500 msec. However, the earlier positive deflection at around 300 msec was visible at the scalp channels but not at the ear channel. The amplitude of the P300 at HC was reduced by 64% in respect to PZ, which corresponds to an amplitude reduction of 4 dB. This reflects that voltage measures are strongly influenced by the spatial distance between channel location and reference location. The effect size of the P300 (expressed as Hedges’ g) was similar for all three channels (Fig.2, bottom) in the time interval of 400–700 msec.

**Figure 3:**
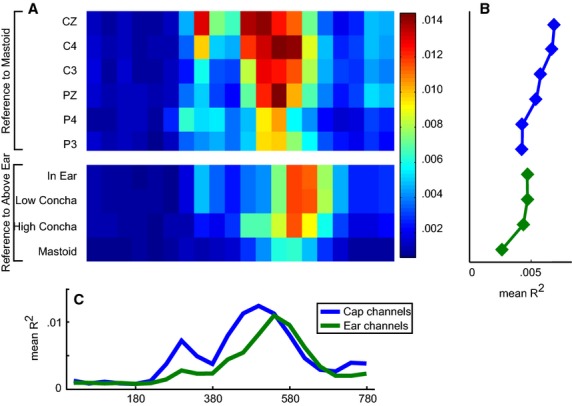
(A) Unsigned *R*^2^ values for individual time bins for cap (top) and ear channels (bottom). (B) Average *R*^2^ over electrodes per time bin for cap and ear channels separately. (C) Average *R*^2^ over time bins per electrode.

The point biserial correlation analysis revealed that the most informative channels in the cap were on average channels CZ and C4, and the most informative time bins were at around 300 and 500 msec after stimulus (Fig.3). For the ear channels, the correlation was slightly lower, with the most informative interval being evident at around 560 msec.

For the online session, 88% of the letters were classified correctly using all electrodes, that is, cap and ear channels. For more than half of the errors made (16 out of 27), the classifier identified the correct row or column, that is, the spelled letter was in the same row or column as the target letter. The accuracy was comparable to the results reported in De Vos et al. (2014b) (Table1). However, the number of flashes was subject dependent in the former study, while it was fixed to 12 flashes in our study, which was reflected in the lower ITR. The number of flashes needed to obtain optimal performance in the training block was 8.9 (SD = 3.1) in the former study and 7.8 (SD = 3.05) in the current study; there was no significant difference between the two studies (*t*-test, *t* (26) = −0.651, *P *= 0.52).

**Table 1:** Comparison of current results with the results reported in De Vos et al. (2014a)

	Current study	De Vos 2014
Paradigm	Identical	
Signal Processing	Identical	
Number of flashes	Fixed (12 flashes per row/column)	Subject dependent (3–12 flashes per row/column)
Electrode locations	C3*, Cz, C4, P3, Pz, P4 mastoid, above ear, in-ear, concha (low, high) Fz (ground), FCz (reference)	FPz, F3, Fz, F4, C3, Cz, C4, TP9, TP10, P3,Pz, P4, O1 and O2; AFz (ground), FCz (reference)
Electrode types	Mini ton electrodes (2 × 4 mm)	Standard ring electrodes
Number of flashes needed for optimal performance in training block	7.83 (SD = 3.05) Range 3–12 flashes	8.9 (SD = 3.1) Range 3–12 flashes
Average classification accuracy (online)	88%	85% (mobile amplifier) 85% (wired amplifier)
Information transfer rate	8.33 bits/minute	10.94 bits/minute (mobile amplifier) 11.34 bits/minute (wired amplifier)

* The electrodes labels according to the international 10–20 system.

## Discussion

We provide here further evidence that miniaturized EEG electrodes allow to record meaningful neural signals. This was shown previously separately for scalp (Nikulin et al. 2010) and ear locations (Kidmose et al. 2012, 2013; Hoon Lee et al. 2014). Our study is the first using identical electrodes for concurrent EEG recordings at both sites, which facilitates a direct comparison. We show for 12 individuals performing a typical online BCI application that meaningful single-trial EEG and trial-averaged ERP can be recorded from scalp and ear sites using miniaturized electrodes.

The results we have obtained are overall comparable to an earlier study using classical EEG electrodes positioned on the scalp with an electrode cap (De Vos et al. 2014b). In contrast to the earlier study, we have used here a fixed number of flashes for all participants, which makes a direct comparison of the current to the previous study's online performance difficult. However, the optimal number of flashes as determined during the training block and the classification accuracy was very comparable between the studies. This underlines the general feasibility of our setup for BCI applications.

The P300 amplitude at the ear was reduced by a factor of two compared to the scalp channels. This amplitude reduction is expected as the distance between the ear electrodes is much smaller compared to the scalp electrodes. However, with the signal amplitude, the noise amplitude was reduced as well, giving rise to similar effect sizes for ear and scalp EEG recording. A reduction in signal amplitude is not detrimental if high-precision amplifiers are used.

More importantly, the information content at the individual channels, as reflected by Hedges' g and the *R*^2^ value, was comparable between cap and ear electrodes, despite the amplitude reduction at the ear channel. This can be explained by the reduced influence of the SSVEP on the ear channels. It is also apparent that the P300 peaked slightly later for the ear channels, which may reflect the different angle between the scalp versus the ear bipolar channel orientation and the P300 neural generators. Future studies have to investigate whether also components less pronounced than the P300 can be recorded reliably.

The mini ton electrodes in combination with the amplifier that was integrated into the rim of the baseball cap allow for concealed and unobtrusive EEG recording. A person wearing a baseball cap (or some other headdress) and/or the ear EEG does not stand out in public, unlike one would with a standard EEG cap. This can help to reduce the perceived stigmatization of wearing an EEG cap and will thereby increase the acceptance of the user for using the EEG both at home as well as in public. This setup further allows to record EEG publicly (e.g., for studying social interactions) with as little interference with normal behavior as possible.

Though there are numerous practical advantages of the ear EEG over scalp EEG (Looney et al. 2011; Hoon Lee et al. 2014), the full potential of exclusively using electrodes in and around the ear remains to be demonstrated. Kidmose et al. (2013) reported SSVEP and ASSR for ear and scalp channels from eight participants as well as AEPs and VEPs for a subgroup of three participants. In contrast to our study, they used different electrodes for the scalp and ear channels. These authors also reported strong amplitude reductions of approximately 20 dB for ear channel compared to scalp channel VEP and AEP amplitudes. This larger amplitude reduction compared to the modest amplitude reduction of approximately 4 dB we found in our study can be explained by the smaller distance of the ear electrodes in the Kidmose study; in contrast to Kidmose, we also used electrodes that are located around the ear. Kidmose and colleagues report that the overall shape of the ERP waveform is comparable between scalp and ear channels. Our data indicate that ear and scalp channels share some waveform characteristics but show clear differences as well. Given the number of different cortical sources to the P300 (Debener et al. 2005; Linden 2005), it is well possible that the ear EEG configuration used in the present study is not sensitive to all neural sources contributing to the P300 complex, due to the orientation and distance of the HC electrode to the reference electrode used. How the factors channel number, electrode distance, and bipolar channel orientation contribute to the sensitivity for recording different brain signals remains to be investigated in future studies.

We have used bipolar electrode derivations in this study. Each ear channel formed a bipolar pair with the electrode above the ear while each cap channel formed a bipolar pair with the mastoid channel. These electrode pairs measure a potential difference and are sensitive to the location and the orientation of the neural sources: A bipolar pair that is located across the isopotential line is sensitive to a given source while a bipolar pair that is placed along the isopotential line is not (Nunez and Srinivasan 2006). The lower influence of the SSVEP on HC compared to PZ is potentially a direct consequence of the orientation and the distance of the electrodes in respect to the underlying source, which for the SSVEP can be expected to be located in the occipital cortex (Di Russo et al. 2007). Future studies will have to investigate the relationship between electrode pair orientations at different ear sites and their sensitivity to different neural processes. A precise acquisition of electrode distances and orientations by means of 3D electrode digitization would be desirable, along with a fine-grained sampling of different bipolar channel angles and distances in and around the ear.

Our primary objective here was to assess the quality of the mini electrodes at different recording locations while keeping other factors constant. As we expected the absolute signal amplitudes at the ear sites to be small (Kidmose et al. 2013), we wanted to assure that the amplitude resolution of the amplifier was sufficient.

The 14-channel wireless EEG amplifier used in our previous mobile EEG studies (e.g., Debener et al. 2012) had insufficient amplitude resolution (0.5 uV) to support adequate ear EEG acquisition; therefore we used a standard laboratory-based amplifier with a better amplitude resolution in the current study. However, modern state-of-the-art mobile EEG amplifiers feature higher precision and should be able to detect the signals at the ear sites also in truly mobile recording conditions.

Future studies have to show the robustness of this recording setup during motion to assure that it is suitable for the noninvasive monitoring of brain activity in daily life situations (see De Vos & Debener, 2014, for a review of this concept). Given our own results (Debener et al. 2012; De Vos et al. 2014a), we expect that recordings during motion should be possible if the following criteria are met: the electrodes are connected firmly with the amplifier, the cables between electrodes and amplifier are as short as possible, amplifier and electrodes move in common and not in isolation, and all electrodes have stable contact with the skin so that the motion of the electrodes in respect to the skin are reduced to a minimum.

The mini electrodes we have used here require a small amount of conductive gel. Arguably dry electrodes would be a more convenient and user friendly way to record EEG (Taheri et al. 1994; Fonseca et al. 2007; Popescu et al. 2007; Gargiulo et al. 2010), but such electrodes do not seem to tolerate movement very well. Currently available EEG dry electrode technology seems not very well suited for capturing high-quality EEG signals while subjects are in motion.

A limiting factor in the present study was the large amount of work, the limited robustness and moderate usability of the self-made ear-pieces. The way the electrodes were placed into the silicone piece made them prone to connection problems. For future applications, a more robust ear-piece design with embedded in-ear and around-ear electrodes will be necessary. By merging ear EEG technology with self-fitting ear-piece technology as developed for hearing protection, it might be possible to manufacture individual EEG ear-pieces without the need of expert intervention. With respect to mobile applications, it also has to be assured that electrodes do not move in the ear canal, even when the mouth moves. The work of Delnavaz and Voix 2014 suggests that it may be possible to identify such a location in the outer ear canal of humans.

## Conclusion

We show here that miniaturized, wet EEG electrodes positioned at discreet scalp and ear locations allow to reliably record ERP signals that are traditionally used in a BCI context. Making EEG recordings nearly invisible by integrating the electrodes either in an unobtrusive head gear or by placing them in and behind the ear promises to be useful for recording EEG on an everyday basis. This will increase user acceptance and it will open up new avenues for the monitoring of human brain functions during daily life situations and actions, out and about.

## Conflict of Interest

None declared.
